# Examining Associations Between Fasting Behavior, Orthorexia Nervosa, and Eating Disorders

**DOI:** 10.3390/nu16244275

**Published:** 2024-12-11

**Authors:** Przemysław Domaszewski, Aleksandra M. Rogowska, Kaja Żylak

**Affiliations:** 1 Institute of Health Sciences, University of Opole, 45-060 Opole, Poland; 2 Institute of Psychology, University of Opole, 45-052 Opole, Poland

**Keywords:** ABOST, anorexia, bulimia, EAT-26, Ramadan, intermittent fasting

## Abstract

Background/Objectives: Fasting, orthorexia nervosa, and eating disorders are increasingly prevalent and interconnected. Understanding their relationship is essential for identifying potential risks and developing effective prevention and intervention strategies. This study investigated these associations to enhance our knowledge of their interplay and implications for mental health. Methods: A cross-sectional online survey was conducted in Poland in 2023. A sample of 214 participants aged 16 to 65 (*M* = 27.95, *SD* = 9.44) participated in this study. Fasting behavior was the predictor (independent) variable, orthorexia nervosa (measured using the Authorized Bratman Orthorexia Self-Test) was the mediator, and an eating disorder was the dependent variable (assessed using the Eating Attitude Test). Results: The Mann–Whitney *U*-test indicated that the fasting group scored higher in orthorexia and eating disorder symptoms than the non-fasting sample. Positive associations emerged between the fasting, orthorexia, and eating disorder scales. Linear regression analysis identified significant predictors of eating disorder symptoms, such as age, fasting, dieting, overweight status, and orthorexia. A path analysis revealed that fasting affected eating disorders directly and indirectly through orthorexia. Conclusions: This study identified fasting as a risk factor for orthorexia and other eating disorders, with orthorexia fully mediating the fasting–eating disorder relationship. Clinicians should consider both fasting and orthorexia when assessing patients at risk for eating disorders. This paper also proposes possible intervention and treatment strategies for affected individuals.

## 1. Introduction

### 1.1. Fasting Behavior

Fasting is a voluntary practice of abstaining from food for a specific period, which holds cultural, spiritual, and medical significance [1]. Different fasting methods offer promising non-pharmacological approaches to improve population health with multiple public health benefits. Fasting encompasses various practices, including Ramadan fasting, intermittent fasting, and Christian Orthodox fasting, each with its unique traditions and practices [2].

Intermittent fasting (IF) encompasses various schedules, each with its unique approach to cycling between eating and fasting periods. The different types of IF schedules are Intra-Daily Fasting (IDF), Whole-Day Fasting (WDF), Delayed Time-Restricted Eating (DTRE), Early Time-Restricted Feeding (ETRF), Alternate-Day Fasting (ADF), Time-Restricted Feeding (TRF), 5:2 and 8:16 diets, Twice-Weekly Fasting (TWF), and Intra-Weekly Fasting (IWF). Complete ADF involves alternating between days of fasting and regular eating [3,4]. The IWF includes ADF and TWF [5]. The most prevalent TRF schedule is the 16:8 fast, which consists of 16 h of fasting and an 8 h eating window [3,4,6,7,8,9,10]. The 5:2 diet includes two days of fasting and five days of regular eating [6,7]. The WDF pattern involves complete fasting for a full day [4]. The IDF involves ETRF and DTRE [6]. Religious fasting, such as Ramadan, involves eating only after nightfall during the month of Ramadan [3].

Different forms of fasting have become common practices for many individuals. It is estimated that approximately 50 million people with diabetes engage in fasting each year [11], and this number does not include healthy individuals who fast to improve their overall health or reduce body fat. Fasting was shown to offer several health benefits for overall health and well-being, including weight loss and metabolic health [8,12,13,14], cardiovascular health [12,15], and several physiological effects [16,17,18]. However, high-quality evidence about the feasibility and long-term outcomes of IF is still lacking [19]. Moreover, fasting was found to be comparable or superior to other dietary interventions for weight management [20]. A study found that time-restricted eating (TRE) reduced body fat and lowered the body mass index in overweight older adults [8,9,10]. It may also provide additional cardiometabolic benefits, such as insulin sensitization, independent of weight loss [21]. While the evidence is inconclusive, fasting was shown to be more effective than an ad libitum dietary intake and equally or more effective than continuous energy restriction for weight management [20]. Combining both fasting and dieting can lead to additional health benefits. Studies showed that a Mediterranean diet intervention with a 5-day semi-fast resulted in reduced total cholesterol, LDL, and body fat, as well as improved insulin sensitivity [22]. A Fasting-Mimicking Diet (FMD) imitates fasting for 4–5 consecutive days, showing alleged beneficial effects on cardio-metabolic risk parameters [23]. Different diets associated with IF can alternate heavy restriction and regular food intake, with variable results on weight and lean body mass loss [6].

### 1.2. Fasting and Eating Disorders

Eating disorders (EDs) involve abnormal eating habits, and individuals with these disorders are concerned about how fasting affects their body shape and weight [24]. The prevalence of eating disorders was 7.8% in 2018, with gender differences (8.4% lifetime ED for women and 2.2% for men) [25]. Fasting is a prevalent behavior associated with eating disorders, with rates increasing in adolescents [26]. However, studies show mixed results regarding fasting’s impact on subsequent eating behaviors. Schaumberg et al. [27] revealed no significant increase in disordered eating behaviors post-fast. The IF can even promote weight loss and improve metabolic health in individuals with obesity [8,9,10,14]. However, risks and side effects should be considered for specific patient groups [13,17,28]. Intermittent fasting, orthorexia, and eating disorders have been associated with body dysmorphic symptoms, with some limitations for specific age and gender groups, especially among women and adolescents [17,29,30,31,32].

Previous research showed that fasting is a risk factor for the development and maintenance of eating disorders [30,33,34,35]. Individuals with eating disorders can be extremely preoccupied with controlling their eating habits, which may include fasting, skipping meals, or other restrictive behaviors. Orthorexia is an obsession with healthy eating, potentially leading to psychological, physical, and social impairments [36,37,38]. The prevalence of orthorexic behavior was observed to be 6.9%, with associations with lower educational attainment, a vegetarian diet, and depressive symptoms [39]. The drive for healthy eating can lead to obsessive thoughts and rigid behaviors about food choices, which align with the harmful symptoms of eating disorders [36]. For individuals with orthorexia, fasting may exacerbate obsessive thoughts and rigid behaviors about food choices, leading to increased psychological distress [36,40]. It is hypothesized that an obsession with healthy eating, combined with fasting as a trend, may contribute to the development of orthorexia. However, longitudinal studies showed that individuals’ risk of engaging in various restrictive eating and other compensatory behaviors decreases over time, with more considerable risk reductions observed over longer follow-up durations [41]. Therefore, older people should experience fewer orthorexic symptoms compared with younger individuals.

Orthorexia symptoms are associated with lower levels of positive eating attitudes and behaviors among college students, indicating a potential negative impact on healthy eating patterns [42]. Several studies indicate a significant association between orthorexia and eating disorders [43,44,45,46,47]. Orthorexia is associated with a higher prevalence of eating disorder symptoms, particularly in individuals with a previous diagnosis of eating disorders [44,45]. Since symptoms of orthorexic and anorexic eating behavior tend to overlap, some scientists suggest that orthorexia nervosa should be classified into the spectrum of eating disorders [43] or even a subtype of anorexia [44]. Research suggests that orthorexia symptoms can predict changes in eating disorder psychopathology and general mental health [48].

### 1.3. The Current Study

**Figure 1:**
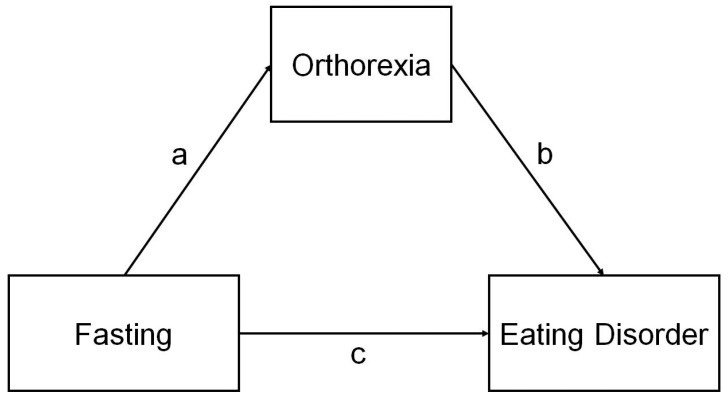
Hypothesized path plot for direct association between fasting and eating disorders (path c) via orthorexia (paths a and b).

While the benefits of fasting are supported by evidence, more research is needed to understand its underlying biological mechanisms and multidimensional effects on health. In addition, although the association between orthorexia and eating disorders is evident, more studies are needed to investigate its relationship with fasting, dieting, overweight, and eating disorders [47]. The impact of fasting on eating behaviors remains unclear, as some studies found no significant increase in disordered eating behaviors following a fast, while others suggested that fasting may trigger or exacerbate disordered eating behaviors, especially in individuals with a history of eating disorders [27]. Therefore, further research is needed to explore the relationships between fasting and eating disorders, particularly orthorexia nervosa. The present study explored for the first time the associations between fasting and orthorexia. While there is no direct link between the two eating behaviors, it can be inferred that orthorexia involves an extreme fixation on healthy nutrition, potentially leading to psychological distress and obsessive-compulsive symptoms. Fasting, on the other hand, is a controlled period of abstinence from food with various cultural and health-related significances. This study aimed to understand how fasting contributes to orthorexia nervosa, taking into account other eating disorders, such as anorexia, bulimia, and overweight. This research will be critical in preparing potential treatment approaches for individuals with orthorexia who engage in fasting behaviors. Based on previous research, we assumed the following:

1. Fasting people score higher compared with non-fasting individuals in terms of symptoms of orthorexia and eating disorders.
2. There is a positive association between fasting, dieting, overweight, orthorexia, and eating disorders symptoms.
3. Fasting contributes to eating disorder symptoms directly (path c) and indirectly via orthorexia (paths a and b) (Figure 1).

## 2. Materials and Methods

### 2.1. Study Design and Procedure

A cross-sectional online survey was conducted in Poland in 2023 using Google Forms. The IRB approved the study protocol. The only inclusion criterion was an age of at least 16 years. This study was anonymous and voluntary, and participants could withdraw from this study at any time if they felt discomfort. No compensation or rewards were offered for participation. Informed consent was obtained from participants before starting this study. Invitations to participate in this study were shared by researchers on private Facebook and Instagram accounts. Researchers asked participants to share the invitation with their friends on these social networking sites. Due to snowball sampling, participants shared and retweeted the invitation.

A sample size was determined using G*Power ver. 3.1.9.7. software. An expected sample size of 176 people (88 individuals in each group) was calculated for Student’s *t*-test, with a moderate effect size (Cohen’s *d* = 0.50), *p* < 0.05, and 0.95 power. In comparison, a desired sample size of 115 people was required for the correlation analysis. Initially, 218 people responded to the invitation, but four individuals refused to participate. Therefore, the total sample included 214 persons. The final sample size was more than adequate for this study’s purpose.

### 2.2. Measures

#### 2.2.1. Eating Patterns Related to Fasting and Dieting

Fasting was defined as “specially planned eating breaks”. Participants responded to questions regarding their fasting behavior by selecting options about their fasting pattern (“How often do you fast?”) and fasting adherence length (“How long have you been fasting?”). Dieting was defined as “specially developed nutritional methods in which selected food products or their quantity are eliminated or limited in order to achieve a specific health effect”. Respondents were asked to choose their current diet (“What diet are you currently following?”) and primary motivation for dieting (“What are the reasons for following this diet?”). The last question concerned health problems related to the introduction of a special diet (“Do you suffer from food allergies or chronic diseases related to the obligation to maintain a specific diet?”).

#### 2.2.2. Eating Disorders

The 26-item Eating Attitude Test (EAT-26) is a self-reported measure developed to identify the presence of the risk of eating disorders (EDs), such as anorexia nervosa, binge-eating disorder, and bulimia nervosa [49,50]. The EAT-26 comprises three subscales: (1) dieting (EAT_D, 13 items characterized by the scrutiny of calorie content, carbohydrates, and sugar content that is motivated by a desire to be thinner); (2) bulimia and food preoccupation (EAT_BFP, six items described by the tendency to purge after meals, as well as excessive food-related thinking), and (3) oral control (EAT_OC, seven items that refer to the tendency toward eating self-control). A global score is calculated by summing all the items. Higher scores indicate more severe eating disorder symptoms, with a score of 20 or above indicating a risk of eating disorders. Participants rate each item on a 6-point Likert scale (3 = *Always*, 2 = *Usually*, 1 = *Often*, 0 = *Sometimes*, *Rarely*, *Never*). In addition to the EAT-26 items, the identification of individuals at risk of eating disorders is based on behavioral questions (with Yes = 1 or No =1 response coding) concerning binge eating (EAT_A), unhealthy shape control (EAT_B), purging (EAT_C), excessive exercise (EAT_D), and significant weight loss in the past six months (EAT_E). The EAT-26 demonstrates good internal consistency in the present study, with a Cronbach’s α of 0.90 for the total score and 0.89, 0.75, and 0.64 for dieting, bulimia and food preoccupation, and oral control, respectively. Individuals in the overweight and obese group reported higher levels of fear of binging, preoccupation with food, desire to be thinner, and dieting behavior than those in the normal weight group [51].

#### 2.2.3. Orthorexia Nervosa

The Authorized Bratman Orthorexia Self-Test (ABOST) was developed by Bratman to assess orthorexia nervosa symptoms and validated in the Polish population [46]. The ABOST includes six statements (e.g., “I spend so much of my life thinking about, choosing, and preparing healthy food that it interferes with other dimensions of my life, such as love, creativity, family, friendship, work, and school.”), rated on a 5-point response scale (ranging from 1 = *Strongly disagree* to 5 = *Strongly agree*). The total score is calculated by summing all the scores, with a higher total score suggesting a greater risk of orthorexia nervosa, with a score of 19 or more indicating a risk of orthorexia [46]. The reliability coefficient Cronbach’s α of 0.83 was found for the ABOST in the present study sample.

#### 2.2.4. Demographics

Participants self-reported a range of demographic characteristics, including age, gender, weight, and height, to calculate their body mass index (BMI; BMI given in kg/m^2^), education, place of residence, and economic status. A BMI < 18.5 was interpreted as underweight, BMI = [18.5–24.9] meant average weight, overweight was identified if BMI = [25.0–29.9], and BMI > 30.0 indicated obesity.

### 2.3. Participant Demographic Characteristics

The sample of 214 people ranged in age from 16 to 65 years old (*M* = 27.95, *SD* = 9.44). The majority of participants were women (87%) with normal weight (67%), a bachelor’s education status (34%), lived in a city with 100,000 to 500,000 inhabitants, and had a self-reported good economic status (51%) (Table 1).

### 2.4. Statistical Analyses

Frequencies of particular categories of sociodemographic variables and eating behaviors were presented using the number of people (*n*) and percentage (%). Since the sample did not meet the criteria for the normal distribution of the data, the Mann–Whitney *U*-test was performed to examine the differences in orthorexia and eating behavior symptoms between fasting and not-fasting individuals. A rank biserial correlation (RBC) was used to assess the effect size, and *p* < 0.05 was assumed to indicate significant intergroup differences.

Associations between fasting, dieting, overweight, and orthorexia and eating disorder symptoms were examined using Spearman’s rho correlation. A hierarchical multiple linear regression was conducted for eating disorder symptoms as a dependent variable, and relevant demographic variables (i.e., age, fasting, dieting, and overweight) in the first step of the analysis, along with orthorexia in the second step, as predictors. The purpose of this hierarchical regression analysis was to examine the individual contribution of orthorexia to the variance in eating disorders while controlling for demographic variables relevant to both these disorders. The path analysis was conducted to examine the mediation model, with the delta method standard errors, bias-corrected percentile bootstrap confidence intervals (with 1000 replications), and maximum likelihood (ML) as estimators. Bootstrapping was employed to provide robust estimates of standard errors and confidence intervals, as this approach is particularly suitable for handling non-normally distributed data by resampling multiple times to improve the accuracy of an effect estimation [52]. The total, direct, and indirect effects were assessed using this method. All statistical tests were performed using JASP ver. 0.18.3.0 software.

## 3. Results

### 3.1. Eating Behaviors in Participants

The frequencies in each category of eating behaviors are presented in Table 2. Among the fasting people (*n* = 98, 46% of the total sample), the majority had fasted for between 1 and 5 years using the 16/8 fasting pattern. Almost one-third of the respondents followed some diet (*n* = 67, 31%). In most cases, it was a vegetarian diet, and a primary motive was to be healthy. The most common health problem among participants was an allergy, which was the health-related reason for the diet (Table 2).

In the sample, 13% met the criteria for orthorexia nervosa, and the same percentage was found for eating disorder symptoms (Table 3). Taking into account additional behavioral questions in the EAT-26, binge eating was reported by 27% of the respondents, weight and shape control was found in 18% of the sample, purging was used by 22% of the participants, excessive exercising was conducted among 8% of the individuals, and significant weight loss during the past year was presented in 12% of the sample (Table 3).

### 3.2. Intergroup Differences in Orthorexia and Eating Disorder Symptoms

As shown in Table 4, the Mann–Whitney *U*-test showed that the fasting group scored higher than the non-fasting sample in symptoms of orthorexia (*p* < 0.01, RBC = −0.24), total score of the EAT-26 test (*p* < 0.01, RBC = −0.23), and its two subscales of dieting (*p* < 0.01, RBC = −0.25) and bulimia and food preoccupation (*p* < 0.05, RBC = −0.18). However, the effect sizes were small for all of these effects. Only the subscale of oral control did not differentiate between the samples of fasting and non-fasting participants (Table 4).

### 3.3. Associations Between Fasting, Dieting, Overweight, and Symptoms of Orthorexia and Eating Disorder

**Figure 2:**
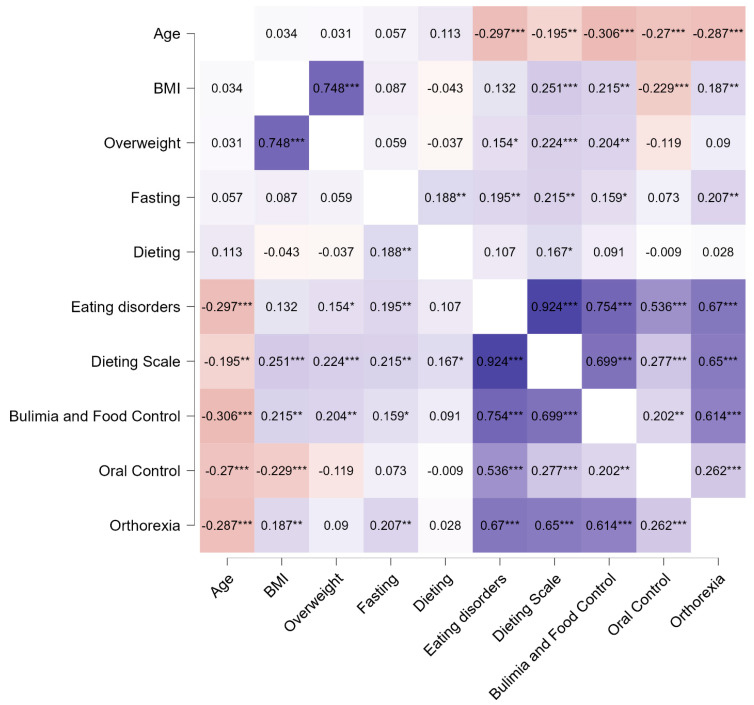
Spearman’s rho heatmap. BMI—body mass index. * *p* < 0.05, ** *p* < 0.01, *** *p* < 0.001.

Positive associations were found between fasting and variables such as dieting, orthorexia, and total score for eating disorders symptoms, as well as its two subscales: (1) bulimia and food preoccupation and (2) dieting (Figure 2).

**Figure 3:**
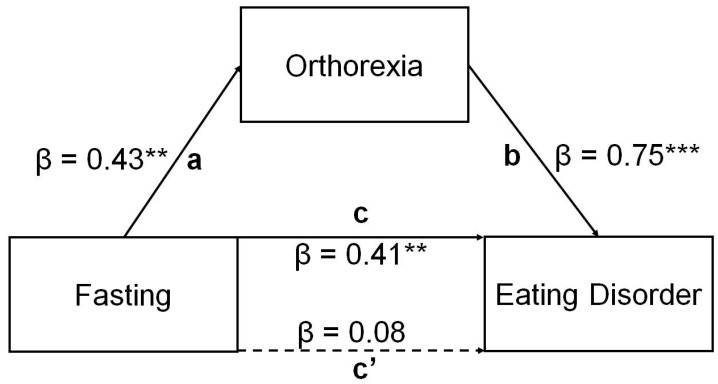
Path plot for the indirect effect of fasting on eating disorder via orthorexia. Path a—effect of fasting on orthorexia; path b—effect of orthorexia on eating disorder; path c—total effect of fasting on eating disorder; path c′—direct effect of fasting on eating disorders in the absence of orthorexia. ** *p* < 0.01, *** *p* < 0.001.

The results of hierarchical multiple linear regression for eating disorder symptoms are presented in Table 5. Age, fasting, dieting, and overweight were significant predictors in the first step of the regression model, and all together explained 19% of the eating disorders behavior. Orthorexia was added to the regression model in the second step to examine changes in the unstandardized regression coefficient (*b*) of fasting behavior and variance in eating behavior, which could be explained selectively by orthorexia symptoms (*R*^2^). When orthorexia was introduced to the regression model, fasting lost its significance, while *R*^2^ increased, indicating the full mediation effect of orthorexia on the relationship between fasting and eating disorders. Orthorexia could solely explain 44% of the eating disorders variance. The path analysis confirmed the full mediation effect of orthorexia on the relationship between fasting behavior and eating disorder symptoms, as reported in Table 6 and Figure 3. To ensure robust results, we used the delta method for standard errors, bias-corrected percentile bootstrap confidence intervals (with 1000 replications), and the maximum likelihood (ML) estimator. These methods are recommended for non-normally distributed data to provide accurate parameter estimates and confidence intervals [53].

## 4. Discussion

For the first time, fasting behavior was examined in the context of variables such as age, BMI, dieting, overweight, orthorexia, and eating disorders. The complex model showed positive associations between fasting and other eating behaviors (both healthy as dieting and unhealthy as orthorexia nervosa and eating disorders), as well as confirmed that orthorexia can fully play a mediating role in the relationships between fasting and eating disorders. Consistent with the first assumption (H1), this study confirmed that fasting individuals scored higher than those non-fasting regarding symptoms of orthorexia and eating disorders. In particular, significant differences were found in the total score of EAT-26 and its two subscales: (1) dieting and (2) bulimia and food preoccupation. Furthermore, fasting behavior positively correlated with dieting, orthorexia, and eating disorders symptoms (especially with the bulimia and food preoccupation and dieting subscales), which confirmed H2.

The present research is consistent with a large body of research, indicating that fasting can be considered a risk factor for the development of eating disorders [13,17,24,27,30,33,34,35,41,54,55]. Fasting has been identified as a risk factor for pathological eating patterns [30,35]. Following a prescribed diet may increase food awareness, potentially leading to eating disorder risk factors that could worsen the therapy of physical illnesses [34]. However, it is important to note that previous studies also showed the ambiguous effects of fasting on eating disorders [14,21]. Therefore, more research is needed to explain these inconsistencies fully.

In particular, IF has been associated with adverse effects and limitations for specific age and gender groups [17]. For example, fasting during Ramadan has been linked to an increase in disordered eating patterns in adolescents, potentially triggering or exacerbating eating pathologies [29]. Furthermore, the relationship between fasting and eating disorder behaviors was found to be consistent, particularly in women, highlighting the potential psychological impact of IF for individuals with a history of eating disorders [30]. Unfortunately, this study was unable to compare fasting behavior across sexes since the vast majority of participants were women (87%). In addition, the group of adolescents and older adults was not representative in this study. Future research should include a larger and more representative group of adolescents and adults for each age group (from early adulthood to older ages), as well as a more gender-balanced group of respondents. Women may engage in fasting more often than men. However, research is needed on a representative sample of the general population, preferably in an international context, because intercultural considerations may have a crucial impact on fasting behavior.

Orthorexia nervosa is characterized by an obsessive focus on “healthy” eating, inflexibility in diet, and clinically significant medical or psychosocial impairment [56]. Research showed several risk factors for orthorexia development. Individuals with a higher severity of orthorexic behaviors tend to be women, have lower age and education levels, report normal weight, have a high frequency of physical activity, have an interest in specific diets, exhibit signs of body image disturbance, and have symptoms of obsessive-compulsive disorders [57].

Although we did not find a direct association between orthorexia and fasting in the previous literature, some researchers suggested that orthorexia may be understood as a type of eating disorder [36,37,38,42,43]. Indeed, studies confirmed a positive correlation between orthorexia and eating disorders [42,43,44,45,47,58]. Moreover, the available evidence suggests that orthorexia can be a predictor of eating disorders, particularly in specific populations, such as student-athletes and individuals with IBS [40,59,60]. Previous studies also found higher orthorexia symptoms in men than women and gender differences in particular variables that predicted orthorexia symptoms [61]. In the present study, we confirmed for the first time that orthorexia was positively related to fasting. Fasting can negatively affect hunger control and increase the temptation to eat unhealthily, as suggested in previous studies [33].

The present findings indicate that age, fasting, dieting, and being overweight contribute to eating disorder symptoms, explaining 19% of this variable. Orthorexia alone explained as much as 44% of the eating disorders variance and fully mediated the relationship between fasting behavior and eating disorders. Orthorexia is associated with overvalued ideas concerning the health-promoting effects of foods, obsessive and ritualized ways of preparing and consuming foods, and illness-anxiety-related thoughts [62]. Previous research showed that individuals with a previous diagnosis of eating disorders and those who followed a restrictive diet or a vegan/vegetarian diet are more likely to exhibit orthorexic tendencies [45]. The current findings showed the potential mechanism of how fasting behavior contributes to eating disorders via orthorexia. Both fasting and orthorexia are types of restrictive behavior related to eating. It is possible that a restriction in fasting behavior, which concerns the time of eating during the day or week and the amount of food, may develop orthorexia by increasing the interest in healthy food and involvement in activities related to eating. Food restrictions can start from fasting and lead to an obsession with healthy eating and, consequently, orthorexia. Such behaviors may also contribute to the development of anorexia and bulimia. On the other hand, obese people may start the fight against excess weight with fasting behavior and progress through obsessive thinking about healthy food to other eating disorders. In this way, the cycle of negative eating behaviors can continue and lead to worsening physical and mental health.

Although the results of these studies are promising, it should be noted that there were a number of limitations that do not allow for the generalization of these studies. First of all, the sample was not very large and balanced in terms of gender and age. Specifically, the small number of male participants suggests that the findings may not fully represent the experiences or behaviors of male individuals. Future studies should be conducted in the general population to determine the prevalence of fasting behavior, mainly due to gender and age. We used self-report measures in an online survey, so we did not control the actual behavior, but the subjective reports of the study participants may be burdened with recall bias. Therefore, experimental studies with more objective measurements are suggested for future research. In addition, the cross-sectional nature of this study makes it impossible to draw confident conclusions about causal effects. We recommend longitudinal studies to verify current research findings.

## 5. Conclusions

This study suggests that fasting people demonstrated higher symptoms of orthorexia and eating disorders than the control sample. Although fasting generally has beneficial effects on physical health, there is a risk of developing eating-related mental health disorders. Doctors and dietitians who recommend fasting to their patients should be aware that it can lead to orthorexia and eating disorders. The issue of attitudes toward a healthy diet and one’s own body should be included in the clinical interview with a patient. If the patient shows features of orthorexia or symptoms of eating disorders, forms of treatment other than fasting should be proposed. Orthorexia was found to be a mediator in the relationship between fasting and eating disorders. We showed that fasting can lead to the intensification of orthorexia symptoms and, in turn, to other eating disorders. However, further research is necessary to fully understand the progression and long-term implications of orthorexia on eating disorders. The present studies can be considered an introduction to longitudinal research.

This study is critical in developing potential treatment strategies for individuals with orthorexia who engage in fasting behaviors. While healthy and balanced eating is essential, obsessive healthy eating fixations may increase the risk for eating disorders, highlighting the need for more education and awareness to minimize the risk for orthorexia and eating disorders. Orthorexia nervosa is characterized by a pathological fixation on food purity and nutrition, and little is known about the risk factors associated with the condition or if the condition should be treated in a similar way to other eating disorders [63]. The overlap between orthorexia and eating disorders suggests the need for a comprehensive approach to treatment, combining interventions from classic eating disorder therapy and nutritional counseling. However, it is essential to note that the available research tools do not sufficiently identify the boundary between an excessive interest in healthy eating and lifestyle and a real disorder that affects everyday functioning. A therapeutic approach that combines interventions and methods from classic eating disorder therapy and nutritional counseling is proposed as a means of treating possible consequences of mental or social distress caused by orthorexia [64].

## Figures and Tables

**Table 1:** Participant demographic characteristics.

Variable	Categories	*n*/*M*	%/*SD*
Age	Years old	27.95	9.44
Gender	Women	185	86.45
	Men	26	12.15
	Nonbinary person	3	1.4
Biometric data	Height	168.82	7.86
	Weight	66.02	13.94
	Body mass index	23.06	3.97
	Underweight	18	8.41
	Average weight	143	66.82
	Overweight	42	19.63
	Obese	11	5.14
Education	Primary	4	1.87
	Professional	3	1.4
	Secondary	65	30.37
	Bachelor’s degree	73	34.11
	Master’s degree or higher	68	31.78
Place of residence	Village	65	30.37
	City with up to 20,000 inhabitants	34	15.89
	City with 20,000–100,000 inhabitants	29	13.55
	City with 100,000 to 500,000 inhabitants	66	30.84
	City with more than 500,000 inhabitants	20	9.35
Economic status	Poor (not enough to meet basic needs)	4	1.87
	Sufficient (enough to meet basic needs)	48	22.43
	Good (slightly more than to meet basic needs)	109	50.94
	Very good (enough to meet all needs)	53	24.77

**Table 2:** Self-reported fasting and dieting behavior.

Variable	Categories	*n*	%
Fasting	No	116	54.21
	Yes	98	45.79
Duration of fasting	I don’t use any fasts	117	54.67
	From a week to two months	21	9.81
	From 2 to 6 months	10	4.67
	From 6 to 12 months	22	10.28
	From 1 to 5 years	28	13.08
	Over 5 years old	16	7.48
Fasting pattern	I don’t use any fasts	117	54.67
	Every day 12/12 h	12	5.61
	Every day 14/10 h	10	4.67
	16/8 h every day	36	16.82
	18/6 h every day	7	3.27
	Every other day	4	1.87
	Once a week	10	4.67
	Twice weekly	5	2.34
	Other pattern	13	6.07
Dieting	No	147	68.69
	Yes	67	31.31
Type of diet	I don’t follow any special diet, but I try to eat healthily	117	54.67
	I don’t follow any special diet, I eat whatever I want	30	14.02
	Meatless diet (vegetarian, vegetarian, vegan, fruitarian, etc.)	22	10.28
	Clean eating diet (no artificial additives, preservatives, sugar, etc.)	15	7.01
	Slimming diet	10	4.67
	Paleo diet (based on meat and animal fats)	3	1.40
	Colorful diet (based on colorful fruits and vegetables)	3	1.40
	Other diets	14	6.54
Motives of dieting	For my health	103	48.13
	I don’t follow any special diet	84	39.25
	Because the doctor recommended it to me	5	2.34
	To avoid unpleasant ailments (e.g., heartburn, stomach pain, headaches)	22	10.28
Health problems	I do not have allergies or other chronic diseases	144	67.29
	Allergy	46	21.50
	Diabetes, insulin resistance	5	2.34
	Reflux, stomach or duodenal ulcers, irritable bowel syndrome, liver disease	12	5.61
	Other reasons (i.e., Hashimoto’s disease, hypothyroidism, high cholesterol, hypertension, low iron, or obesity)	7	3.27

**Table 3:** Self-reported eating disorder symptoms.

Variable	Categories	*n*/*M*	%/*SD*
Orthorexia test	ABOST total score (*M*, *SD*)	12.08	5.55
Orthorexia symptoms	No (ABOST < 19)	187	87.38
	Yes (ABOST > 18)	27	12.62
Eating disorder test	EAT-26 total score (*M*, *SD*)	15.96	12.69
	Dieting subscale (*M*, *SD*)	10.17	8.11
	Bulimia and food preoccupation subscale (*M*, *SD*)	2.37	3.50
	Oral control subscale (*M*, *SD*)	3.42	3.39
Eating disorders symptoms	No (EAT-26 < 20)	187	87.38
	Yes (EAT-26 > 19)	27	12.62
Binge eating	No (EAT_A = 0)	157	73.36
	Yes (EAT_A = 1)	57	26.64
Shape control	No (EAT_B = 0)	175	81.78
	Yes (EAT_B = 1)	39	18.22
Purging	No (EAT_C = 0)	168	78.51
	Yes (EAT_C = 1)	46	21.50
Excessive exercise	No (EAT_D = 0)	197	92.06
	Yes (EAT_D = 1)	17	7.94
Weight loss	No (EAT_E = 0)	188	87.85
	Yes (EAT_E = 1)	26	12.15

**Table 4:** Mann–Whitney *U*-test for orthorexia and eating disorder symptoms by fasting.

Variable	Not Fasting (*n* = 98)		Fasting (*n* = 116)		*U*-Test	*p*	RBC
	*M*	*SD*	*M*	*SD*			
Orthorexia symptoms	10.99	4.86	13.37	6.05	4323.50	0.002	–0.24
Eating disorder symptoms	13.60	10.99	18.77	14.00	4404.00	0.005	–0.23
Dieting	8.41	6.74	12.27	9.07	4271.50	0.002	–0.25
Bulimia and food preoccupation	1.95	3.28	2.88	3.70	4688.00	0.021	–0.18
Oral control	3.24	3.39	3.62	3.38	5205.00	0.285	–0.08

Note: RBC—rank biserial correction as an effect size.

**Table 5:** Hierarchical multiple linear regression results for eating disorder symptoms.

Model	Variable	*b*	*SE b*	β	*t*	*R*	*R* ^2^	*F*	*df*
H_0_	Intercept	23.03	2.56		8.99 ***	0.44	0.19	12.36 ***	4.209
	Age	–0.44	0.09	–0.32	–5.12 ***				
	Fasting	4.22	1.61		2.62 **				
	Dieting	5.73	1.75		3.27 **				
	Overweight	5.50	1.83		3.00 **				
H_1_	Intercept	–0.82	2.30		–0.36	0.79	0.63	71.03 ***	5.208
	Age	–0.19	0.06	–0.14	–3.13 **				
	Fasting	0.54	1.12		0.49				
	Dieting	4.25	1.19		3.56 ***				
	Overweight	3.87	1.25		3.10 **				
	Orthorexia	1.61	0.10	0.71	15.73 ***				

Note: ** *p* < 0.01, *** *p* < 0.001.

**Table 6:** Parameter estimates for the mediating effect of orthorexia on the relationship between fasting and eating disorders.

								BCa 95% CI	
Effect	Predictor	Mediator	Outcome	*b*	*SE b*	*z*	*p*	LL	UL
Total	Fasting	=>	Eating disorders	5.17	1.70	3.04	0.002	2.01	8.64
Path	Fasting	=>	Orthorexia	2.38	0.74	3.20	0.001	0.76	3.84
Path	Orthorexia	=>	Eating disorders	1.72	0.10	16.70	<0.001	1.48	1.96
Indirect	Fasting	Orthorexia	Eating disorders	4.10	1.30	3.14	0.002	1.33	6.78
Direct	Fasting	Orthorexia	Eating disorders	1.08	1.15	0.94	0.349	–1.18	3.27

Notes: Delta method standard errors and maximum likelihood (ML) estimator; BCa—bias-corrected percentile bootstrap; CI—confidence intervals; LL—lower level; UL—upper level.

## Data Availability

The original contributions presented in this study are included in the article. Further inquiries can be directed to the corresponding author.
